# 844. Landscape Analysis to Identify Effective Drug Repurposing Candidates for the Treatment of Implantation Mycoses: Comparison of World Health Organization Survey Treatment Data and Published Case Reports on CURE ID

**DOI:** 10.1093/ofid/ofad500.889

**Published:** 2023-11-27

**Authors:** Reema Charles, Barbara Milani, Daniel Argaw Dagne, Bisma Ali, Heather Stone, Marco Schito, Raghavendra Tirupathi, Nalini Oliver

**Affiliations:** FDA, Silver Spring, Maryland; WHO, Geneva, Geneve, Switzerland; WHO, Geneva, Geneve, Switzerland; CURE Drug Repurposing Collaboratory, Phoenix, Arizona; FDA, Silver Spring, Maryland; CURE Drug Repurposing Collaboratory, Phoenix, Arizona; Keystone Health, Chambersberg, Pennsylvania; CURE Drug Repurposing Collaboratory, Phoenix, Arizona

## Abstract

**Background:**

Implantation mycoses are a group of fungi that gain access through cutaneous or mucosal wounds or by contact. The most widespread implantation mycosis is sporotrichosis. Chromoblastomycosis, eumycetoma, and coccidioidomycosis are among the others. They are associated with significant morbidity, disability and lack effective and cost-effective therapies.

**Figure:**
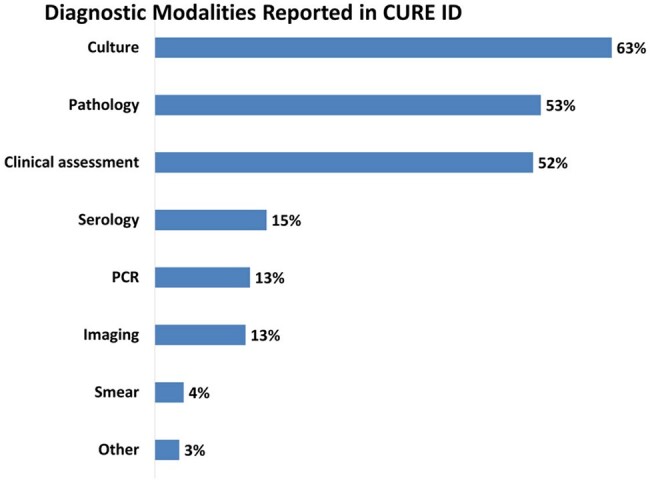

Implantation mycoses are difficult to be diagnosed early, can be recalcitrant and very difficult to treat. In CURE ID database, clinical assessment was reported in 52% of published reports, with culture (bacterial/fungal) being the most reported (63%) diagnostic modality.

**Figure:**
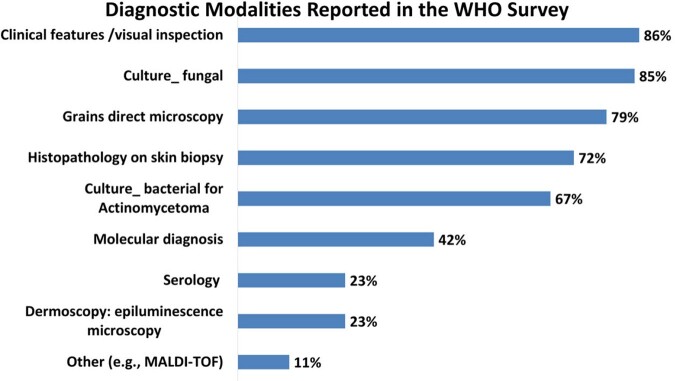

Clinical features/visual inspection (86%) was reported most frequently as a diagnostic tool in the WHO survey.

**Methods:**

World Health Organization (WHO) together with the U.S. Food and Drug Administration (FDA) and CURE Drug Repurposing Collaboratory (CDRC) developed an online survey in early 2022 to capture treatment data on implantation mycoses from around the world.

CURE ID, a web-based repository developed by the FDA and the National Center for Advancing Translational Sciences (NCATS) to gather real-world data (RWD) on the novel uses of existing drugs for difficult-to-treat infectious diseases.

CURE ID team extracted data from all published cases in PubMed. We analyzed 656 published implantation mycoses cases, including sporotrichosis (n=255), chromoblastomycosis (n=198), and eumycetoma (n=46).

**Figure:**
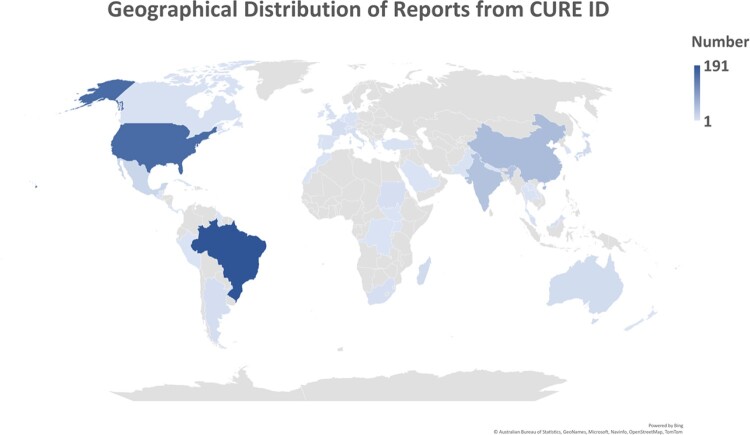

CURE ID case reports are reported from 48 countries, with majority of cases being reported from Brazil (29%).

**Figure:**
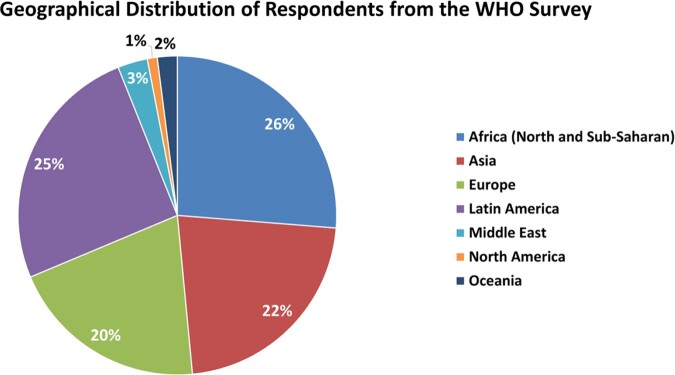

The WHO global online survey collected information from a total of 142 respondents from 47 countries. Majority of the respondents of the survey were clinicians (39%), followed by dual professional profile of laboratory and clinical experts (36%), laboratory technicians/specialists (9%), public health specialists (7%), and others (9%).

**Results:**

For eumycetoma, chromoblastomycosis and sporotrichosis, itraconazole was reported most frequently (85-90%) and terbinafine for refractory cases (44-56%) in the WHO survey. Newer generation azoles (posaconazole and voriconazole) were also reported (27-41%) for chromoblastomycosis and eumycetoma. Flucytosine (14%) and imiquimod (11%) were also reported for chromoblastomycosis. For sporotrichosis, 44% respondents reported using potassium iodide.

Treatment data from CURE ID reports also identified itraconazole as the most frequently used drug across all implantation mycoses. Antidulafungin (2%) is another drug that was reported for sporotrichosis in CURE ID. Other drugs reported for chromoblastomycosis were terbinafine (37%), imiquimod (9%), griseofulvin (6%) and naftifine (3%).

**Figure:**
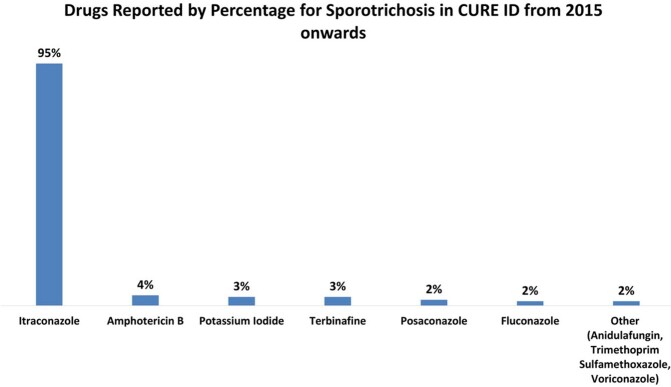

Analyzing treatment data from published case reports on CURE ID from 2015 onwards, itraconazole was also the most frequently used drug across all implantation mycoses. It was used in 95% reports on sporotrichosis, while for eumycetoma and chromoblastomycosis, its use was reported at 64 and 55% respectively. Terbinafine has been reported in 7% cases of eumycetoma and 37% for chromoblastomycosis, while it was used sparingly in just 3% of cases of sporotrichosis. Use of ketoconazole was reported sparingly (1%) for chromoblastomycosis in case reports published from 2015 onwards, while it is reported more consistently for chromoblastomycosis (6%) and eumycetoma (7%) from all the published reports in CURE ID dating back to the 1980s. Use of imiquimod was reported in 9% of cases of chromoblastomycosis. Three percent of reports from CURE ID on sporotrichosis used potassium iodide.

**Figure:**
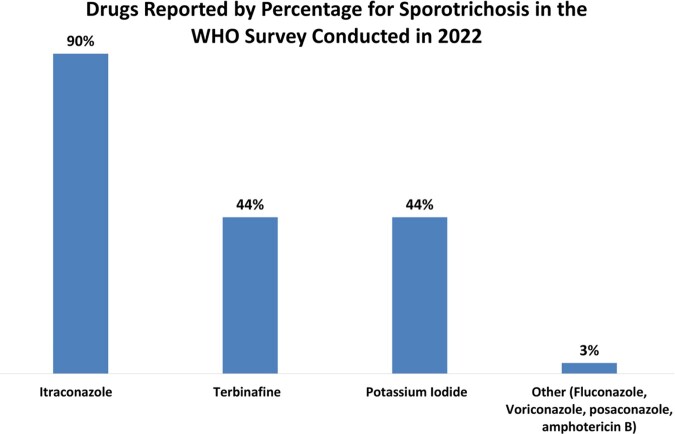

The WHO survey confirms a widespread use of repurposed drugs, with respondents outlining a few medicines that were not included in the survey list. For eumycetoma, chromoblastomycosis and cutaneous sporotrichosis, itraconazole was reported as the first choice (85-90%) and terbinafine for refractory cases (44-56%) in the WHO survey. For chromoblastomycosis, there is a lower but consistent use of flucytosine (14%) and imiquimod(11%). For cutaneous sporotrichosis, we noted a considerable use of potassium iodide (44%).

**Conclusion:**

While itraconazole is already used as a standard of care, it is not yet approved by the FDA for use in these conditions of interest. This is an area of high unmet need and merit a systematic approach to capture large volumes of RWD for potential efficacy signals and hypothesis generation to inform clinical trials.

**Disclosures:**

**All Authors**: No reported disclosures

